# Croton Chloral in Dentistry

**Published:** 1877-06

**Authors:** C. J. Cleborne

**Affiliations:** Naval Hospital, Portsmouth, N. H.—Med. Record.


### ARTICLE XI.

### *Croton Chloral in Dentistry.*

The specific effect of croton chloral hydrate upon the *fifth* pair in neuralgia suggested its use as an internal and local anæsthetic in toothache. I have derived prompt relief from its use in 5-10 grain doses in the toothache of dyspepsia and pregnancy, and in those aggravating cases which occur in rheumatic and neuralgic patients. Failing to relieve by the ordinary remedies a severe attack of toothache, due to dental caries, I found that a mixture of equal parts of crystallized carbolic acid, croton chloral hydrate and solid Japanese oil of peppermint, promptly removed the pain and obtunded all sensitiveness during the process of filling. I suggested the use of this compound to my dentist, who assures me that he has found it to be the "obtunder" he has used for deadening the pain of sensitive dentine, and for removing soreness of the gums, which sometimes results from filling and other operations upon the teeth. In toothache, after the mouth has been washed out with a solution of bicarbonate of soda in warm water, and the cavity of the tooth dried with a pledget of cotton-wool, a small piece of the latter should be soaked in the above mixture—gently pressed to remove excess of moisture—introduced into the cavity, and covered with a small piece of dry cotton-wool; or the latter may be first dipped into styptic-colloid or collodion, and then lightly pressed home. In filling teeth, when it is not necessary nor expedient to destroy the pulp by arsenic or other escharotic, nearly all sensitiveness may be allayed, during the preparation of the cavity, by the following plan: Arm a fine brooch with a small ball of cotton wool, dip it into the mixture (removing any superfluous material by a little pressure,) introduce it into the dental cavity, and retain in position by the introduction of a second piece of wool. In the course of five or ten minutes the cotton-wool may be removed, and the excavation of the cavity may be proceeded with until it again becomes sensitive, when the application should be renewed, and re peated as often as it may be necessary until the cavity is ready for the plug; a result which may be accomplished with little or no pain, and without injury or destruction of the nerve.

C. J. Cleborne, M. D.

Naval Hospital, Portsmouth, N. H.—*Med. Record.*

